# A function-based mapping of sensory integration along the cortical hierarchy

**DOI:** 10.1038/s42003-024-07224-z

**Published:** 2024-11-29

**Authors:** Wei Wei, R. Austin Benn, Robert Scholz, Victoria Shevchenko, Ulysse Klatzmann, Francesco Alberti, Rocco Chiou, Demian Wassermann, Tamara Vanderwal, Jonathan Smallwood, Daniel S. Margulies

**Affiliations:** 1 Cognitive Neuroanatomy Lab, Université Paris Cité, INCC UMR 8002, CNRS, Paris, France; 2 Wellcome Centre for Integrative Neuroimaging, FMRIB, Nuffield Department of Clinical Neurosciences, University of Oxford, Oxford, United Kingdom; 3 Max Planck School of Cognition, Leipzig, Germany; 4 Wilhelm Wundt Institute for Psychology, Leipzig University, Leipzig, Germany; 5 School of Psychology, University of Surrey, Surrey, United Kingdom; 6 Université Paris-Saclay, Inria, CEA, Palaiseau, France; 7 Department of Psychiatry, University of British Columbia, Vancouver, Canada; 8 BC Children’s Hospital Research Institute, Vancouver, Canada; 9 Department of Psychology, Queen’s University, Kingston, Canada

**Keywords:** Sensory processing, Cognitive neuroscience, Computational neuroscience

## Abstract

Sensory information mainly travels along a hierarchy spanning unimodal to transmodal regions, forming multisensory integrative representations crucial for higher-order cognitive functions. Here, we develop an fMRI based two-dimensional framework to characterize sensory integration based on the anchoring role of the primary cortex in the organization of sensory processing. Sensory magnitude captures the percentage of variance explained by three primary sensory signals and decreases as the hierarchy ascends, exhibiting strong similarity to the known hierarchy and high stability across different conditions. Sensory angle converts associations with three primary sensory signals to an angle representing the proportional contributions of different sensory modalities. This dimension identifies differences between brain states and emphasizes how sensory integration changes flexibly in response to varying cognitive demands. Furthermore, meta-analytic functional decoding with our model highlights the close relationship between cognitive functions and sensory integration, showing its potential for future research of human cognition through sensory information processing.

## Abstract (web summary)

A two-dimensional framework representing integrative sensory information along a hierarchy from unimodal to transmodal regions distinguishes between brain states and highlights how sensory integration adapts flexibly to varying cognitive demands.

## Introduction

The human brain operates through a systematic collaboration of modules that are hierarchically organized in a complex system. A global hierarchy extending from the primary to association cortex is a fundamental organizing principle, and has been supported by anatomical^1–6^ and functional^7,8^ evidence. Notably, the primary sensory cortex exhibits higher neuronal density^1^ and well-defined cytoarchitectural layers compared to higher-order regions^2^, as revealed by histological studies. Investigation of the cortical microstructure further indicates a decline in myelin content^3,4^ and cortical thickness^5,6^ from primary to association areas. Recent functional research also highlights a gradient that situates brain areas in an order ranging from unimodal to transmodal regions, similar to anatomical findings. One study was based on functional connectivity leveraging primary sensory seeds^7^, and the other analyzed the principal component derived from the resting-state functional connectome^8^.

This unimodal-to-transmodal hierarchy serves as a framework for signal transmission^9–11^. As signals propagate from primary to higher-order areas, representations become increasingly abstract^12–14^. This progression compresses sensory information into a condensed, less detailed, and multimodal integrated form^15–18^, aligning with an early model of sensory processing organization proposed by Mesulam^19^. According to this model, sensory inputs undergo an abstraction process as unimodal information converges toward the transmodal cortex. This process has been suggested to be linked to the emergence of higher-order cognitive functions^20^, which makes mapping sensory information integration along the processing hierarchy a promising means to elucidate the intricate relationship between cognition and sensory processing.

Frameworks related to the sensory integration process have been posited. Huntenburg and colleagues proposed an intrinsic coordinate system through relative geodesic distance from primary sensory landmarks^21^, which showed an organizational framework for convergent sensory gradients. The study of HCP’s multi-modal parcellation^22^ displayed a visualization framework relevant to sensory integration via functional associations with early sensory areas. However, a more quantitative measurement grounded in functional characteristics could build upon these conceptualizations. While resting-state fMRI has conventionally been employed to investigate intrinsic functional characteristics, recent research using a naturalistic movie-watching paradigm identified distinct principal components that more effectively differentiate sensory systems^23^. This discrepancy may be attributed to the rich coordinated external visual and auditory inputs inherent in movie-watching, providing a potential advantage in characterizing sensory processing streams.

The main objective of this study is to develop a function-based model to systematically map sensory integration along the processing hierarchy. In pursuit of this aim, we evaluate the capability of the proposed sensory integration model to delineate known sensory processing streams, discern diverse states, and characterize functional associations. By applying this model to data collected while participants are processing dynamic, naturalistic stimuli, we seek to advance our understanding of the relationship between sensory integration and higher-order cognitive functions.

## Results

### Sensory integration model

**Fig. 1:**
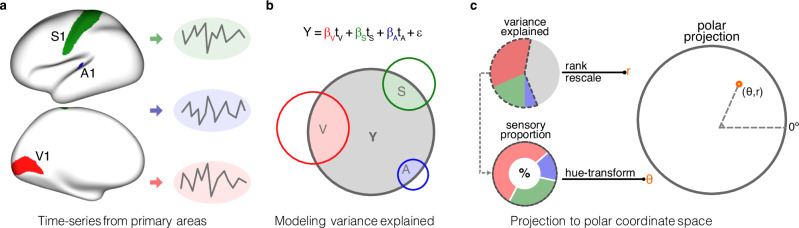
Pipeline to construct the sensory integration model. **a** Mean time series was computed separately for V1, S1, and A1 based on Glasser’s MMP parcellations. **b** A non-negative linear model was used to generate sensory components within each vertex by using primary sensory time series as predictors. The lower Venn diagram provides a schematic of the different components within the above equation. **c** Ratios of the variance explained by primary sensory predictors were ranked and rescaled to a range from 0 to 1, representing one dimension of the sensory integration model, and were named magnitude (r). For each vertex, three sensory parameters (β_V_, β_S_, and β_A_) were converted into an angle (θ) using hue transformation, representing the other dimension that indicated the proportional contributions of different sensory modalities.

We characterized sensory integration along the cortical hierarchy through two dimensions: sensory angle and sensory magnitude (Fig. 1). Sensory angle is calculated by converting three primary sensory associations into an angle. Sensory magnitude is determined by ranking the percentage of variance explained by primary sensory signals.

**Fig. 2:**
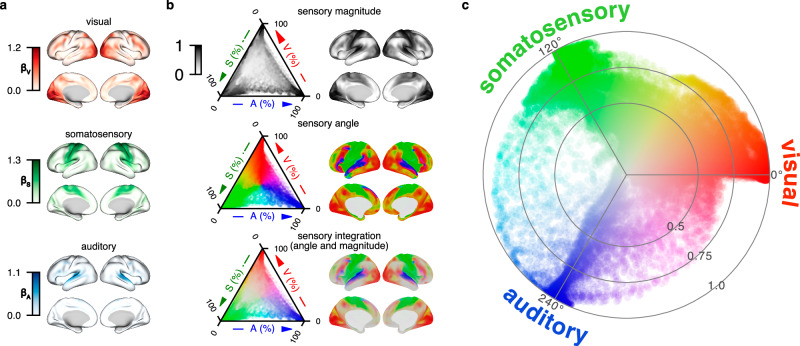
Relationship between sensory parameters and sensory integration model. **a** Surface mapping of group-averaged sensory parameters under movie-watching state. The top row is the visual parameter (β_V_), the middle is the somatosensory parameter (β_S_), and the bottom is the auditory parameter (β_A_). **b** Ternary plots depict the relationship between the proportions of sensory parameters and the sensory integration model under movie-watching state, whereas surface plots display the corresponding spatial locations on the cortical surface. The color representation in the top row is determined by the group-level sensory magnitude. The color in the middle row is derived by applying the group-level sensory angle as hue. The color in the bottom row combines the group-level angle as hue with the group-level magnitude as saturation. **c** Projection of group-level sensory magnitudes and sensory angles under movie-watching state onto a polar coordinate system. The color scheme is identical to that used in the bottom row of panel b.

By fitting primary sensory signals into a linear regression model to explain the time series across the cortical surface, the regression coefficients, referred to as “sensory parameter”, indicate the associations with primary sensory signal. The distributions of these three sensory parameters on the cortical surface reveal gradients of decreasing strength from each primary sensory area toward association regions (Fig. 2a). To illustrate the relationship between sensory parameters and the two dimensions of our model, we visualized the sensory magnitude and sensory angle using ternary plots (Fig. 2b), which graphically depict the proportions of the three sensory parameters as positions within an equilateral triangle. The color coding of the top ternary plot in Fig. 2b is based on the group-level magnitude under movie-watching state, with brighter colors indicating lower magnitudes. The color coding of the middle ternary plot in Fig. 2b is determined by assigning the group-level angle as hue, with the saturation of 1 and brightness of 0.86. Sensory magnitude and sensory angle together represent sensory integration, depicted in the bottom row of Fig. 2b, where the color is determined by using the group-level angle as hue, the group-level magnitude as saturation, and a fixed brightness of 0.86.

The distribution of sensory magnitudes (Fig. 2b, top) exhibits that higher values tend to be situated closer to the triangle corners, while lower values tend to be placed in the inner part of the triangle. The higher magnitude indicates greater variance explained by primary sensory signal. The triangle corners are located at the end of the respective sensory parameter axis and with the highest sensory parameter proportion, which indicates that they are corresponding to primary sensory areas. The surface map using the same color coding of the ternary plot shows the same trend, where higher-level areas with lower magnitudes and brighter color while lower-level areas with higher magnitudes and darker color. The distribution of angles (Fig. 2b, middle) demonstrates that the color with hues close to sensory anchoring angles (0° or red for visual, 120° or green for somatosensory, 240° or blue for auditory) are dominant at the triangle corners and the mixture of colors aligns with the sensory parameter proportions.

For presenting the combination of the two dimensions in our model, we employed a color coding with sensory angle as hue and sensory magnitude as saturation (Fig. 2b, bottom). To visualize magnitude and angle naturally, we projected the two dimensions of our model into a polar coordinate space (Fig. 2c). In this representation, the angle indicates the proportional contributions of three sensory components at each vertex. Visual, somatosensory, and auditory domains are centered at 0°, 120°, and 240°, respectively. For example, if the somatosensory proportion is dominant, its angle ranges from 60° to 180°. When visual proportion exceeds auditory, the angle falls between 60° and 120°, closer to the visual domain. A larger difference between visual and auditory proportions brings the angle closer to 60°. The magnitude reflects the primary sensory associations. Lower magnitude means a lower saturation (or higher grayness) on the surface, and a more central location in the polar coordinate space.

**Fig. 3:**
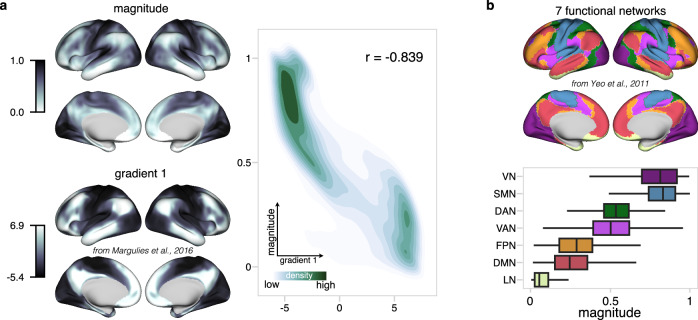
Sensory magnitude and cortical hierarchy. **a** Left column shows surface mappings of sensory magnitude under movie-watching condition and the principal connectome gradient under resting-state condition^8^. The right density illustrates the relationship between sensory magnitude and principal gradient values. **b** The boxplot illustrates the network-wise distribution of the magnitude values based on a 7-network parcellation^24^. For the boxplot, the middle line represents the median, while the box’s upper and lower limits correspond to the 75th and 25th percentiles, respectively. The whiskers extend up to 1.5 times the interquartile range from the upper and lower box limits. Note the striking topographical consistency between the magnitude map and the principal gradient map, despite methodological difference, indicating converging evidence.

The cortical hierarchy spans from primary to high-order areas. As one moves higher in this hierarchy, there is a decrease in association with primary regions, which corresponds to a lower percentage of variance explained by primary sensory signals. One dimension of our sensory integration model, sensory magnitude, was derived by ranking the percentage of primary sensory explained variance across different cortical areas. We hypothesized that sensory magnitude might correspond to the cortical hierarchy. To examine this relationship, we compared sensory magnitude with the principal gradient^8^, a known metric that captures cortical hierarchy based on the resting-state functional connectivity. We illustrated the principal gradient alongside sensory magnitude on the cortical surface (Fig. 3a, left). Additionally, a two-dimensional density plot was used to visualize the relationship between the principal gradient and the magnitude, showing similar trends and a dense overlap at both extremes (Fig. 3a, right). A strong inverse correlation of -0.839 was observed between the principal gradient and sensory magnitude, suggesting that areas higher in the cortical hierarchy have lower sensory magnitude. Furthermore, sensory magnitude was categorized into seven functional networks^24^ (Fig. 3b), and the order presented in the boxplot aligns with the previously observed hierarchical organization^8^. These findings suggest that sensory magnitude effectively reflects the sensory processing hierarchy,

### Test-retest reliability

**Fig. 4:**
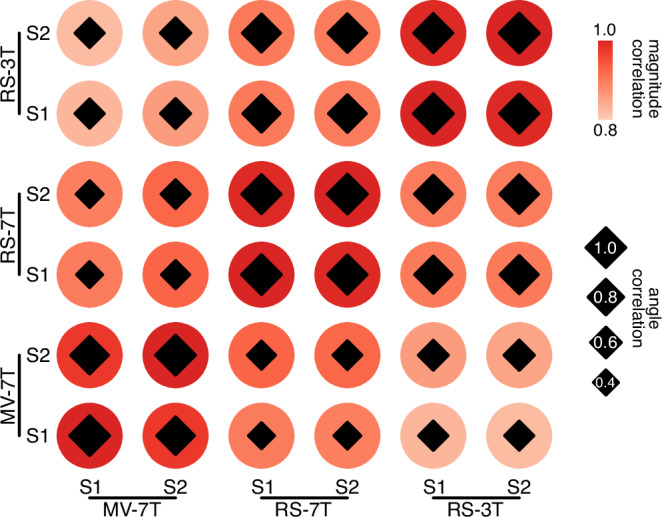
Between-session correlations in magnitude and angle. The size of the diamond within the red circle represents the between-session correlation of angles, with larger diamonds meaning higher correlations. The color of the circle represents the between-session correlation of magnitude, the redder the color, the higher the correlation.

This study included three kinds of functional data (movie-watching at 7 T, resting-state at 7 T and 3 T) from the same participants. Each data type consists of four scans, divided into two concatenated sessions. The test-retest reliability was assessed by calculating the correlation between these sessions (Table 1, Fig. 4).

**Table 1:** Between-state and between-session correlations

Between-state correlations in sensory magnitude			
	MV-7T	RS-7T	RS-3T
MV-7T	1		
RS-7T	0.952	1	
RS-3T	0.914	0.946	1
**Between-state correlations in sensory angle**			
	MV-7T	RS-7T	RS-3T
MV-7T	1		
RS-7T	0.477	1	
RS-3T	0.558	0.907	1

Between-session correlations in sensory magnitude						
	S1-MV-7T	S2-MV-7T	S1-RS-7T	S2-RS-7T	S1-RS-3T	S2-RS-3T
S1-MV-7T	1					
S2-MV-7T	0.985	1				
S1-RS-7T	0.941	0.957	1			
S2-RS-7T	0.939	0.955	0.996	1		
S1-RS-3T	0.901	0.918	0.943	0.941	1	
S2-RS-3T	0.896	0.913	0.943	0.941	0.996	1
**Between-session correlations in sensory angle**						
	S1-MV-7T	S2-MV-7T	S1-RS-7T	S2-RS-7T	S1-RS-3T	S2-RS-3T
S1-MV-7T	1					
S2-MV-7T	0.893	1				
S1-RS-7T	0.429	0.504	1			
S2-RS-7T	0.458	0.529	0.965	1		
S1-RS-3T	0.542	0.570	0.868	0.894	1	
S2-RS-3T	0.537	0.582	0.916	0.938	0.962	1

*S1* session 1, *S2* session 2, *MV* movie-watching, *RS* resting-state.

As detailed in Table 1 and illustrated in Fig. 4, the highest correlations for both angles and magnitudes were observed within the same brain state. The correlations of magnitude were high across brain states and scanning conditions. However, the correlations of angles demonstrated high values only within the same brain state. It is worth noting that the correlations are generally high between test and retest sessions within every data type, indicating the robustness of magnitude and angle.

### Spatial patterns along sensory streams

**Fig. 5:**
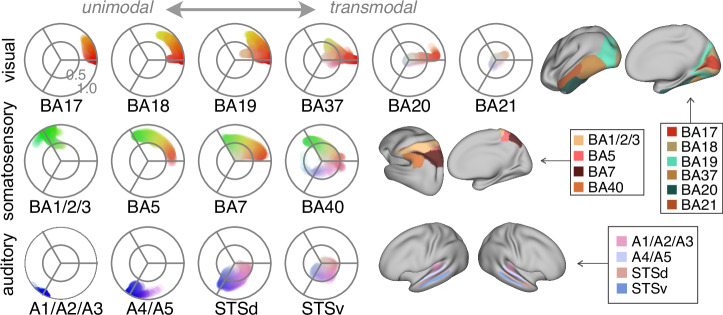
Altering spatial patterns of the sensory integration model along sensory streams. The top row is the visual stream from lower- to higher-level regions, as shown on the cortical surfaces. The middle and bottom rows are somatosensory and auditory streams respectively. BA, Brodmann area; STSd, dorsal superior temporal sulcus; STSv, ventral superior temporal sulcus.

To validate the capability of our model to capture known sensory processing streams, we examined the distribution of patterns within our polar coordinate space along three sensory streams (Fig. 5). The overarching trend entailed a shift from the periphery to the core, indicating that the signal propagates upstream along the unimodal-to-transmodal hierarchy. In addition, the angular distribution of the pattern aligned with the sensory-specific functions of the respective regions. For instance, within the somatosensory processing stream, Brodmann area 5 (BA5 or superior parietal lobule), responsible for somatosensory input and visually-guided grasping, exhibited a spatial pattern across somatosensory (green) and visual (red) domains. Brodmann area 7 (BA7), implicated in visuo-motor coordination, displayed a pattern across somatosensory and visual domains.

Higher-order regions also manifested activity patterns consistent with their functional profiles. Thus, Brodmann area 40 (BA40 or supramarginal gyrus), functioning as a somatosensory association area, is also involved in language perception^25^ and phonological decision making^26^. Its functional profile is commensurate with its angular range spanning all three domains. Likewise, at the highest hierarchical levels within the visual stream, Brodmann area 21 (BA21 or middle temporal gyrus) is linked to audio-visual emotional recognition and semantic comprehension while reading^27^. Its pattern, while centrally located, extended into visual and auditory domains more than somatosensory. A similar pattern was observed at the highest levels of the auditory stream. Specifically, the dorsal and ventral superior temporal sulci (STSd and STSv), that are known for their roles in phonological awareness and audio-visual integration^28^, also demonstrated a centrally located pattern. However, in comparison to BA21, the auditory and visual components extended further into both auditory and visual domains. Notably, the STSd showed more auditory contribution than STSv, reflecting its relative proximity to auditory regions within the STS. Taken together, this polar coordinate space appears to capture intriguing features of the three processing streams that are consistent with their functional roles in their respective sensory processing hierarchies.

### Between-state comparison (movie-watching vs. resting-state)

The human brain operates as a highly adaptive and context-sensitive organ. The ever-changing landscape of brain activity corresponds to specific patterns of neural activation under distinct cognitive states. The dynamic nature of brain states underscores the intricate interplay between brain regions and their functional roles and emphasizes its capacity to modulate the contribution of various regions to cognitive processes depending on the environmental, cognitive, or emotional context. To investigate the capability of the sensory integration model to capture the state-dependent shifts and how sensory integration contributes to cognitive processing under different brain states, we performed a comparison between movie-watching and resting-state.

**Fig. 6:**
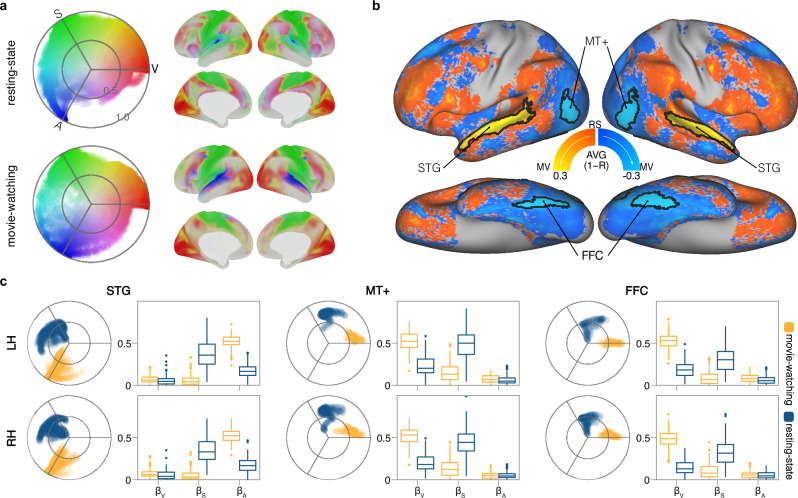
Between-state comparison of sensory angles (movie-watching vs. resting-state). **a** Polar and surface mapping of the sensory integration model under the resting-state and movie-watching condition. The labels V, S, and A mark the anchoring angles for visual (0°), somatosensory (120°), and auditory (240°) modalities. **b** Group-averaged difference of sensory angles between movie-watching and resting-state, the clusters with black outlines were the significantly different areas. Warm colors indicate the angle during movie-watching changes anticlockwise relative to the angle of resting-state, cool colors mean the opposite. **c** Polar plots display the distribution patterns of the significantly different areas between two brain states, while box plots show the comparisons of the averaged sensory parameters within each area. For the boxplots, the middle line represents the median, while the box’s upper and lower limits correspond to the 75th and 25th percentiles, respectively. The whiskers extend up to 1.5 times the interquartile range from the upper and lower box limits. Any data points beyond the whiskers are considered outliers and plotted as individual points. MV movie-watching, RS resting-state, STG superior temporal gyrus, FFC fusiform facial complex, MT middle temporal, LH left hemisphere, RH right hemisphere, β_V_ visual sensory parameter, β_S_ somatosensory sensory parameter, β_A_ auditory sensory parameter.

The two dimensions of the sensory integration model for both resting-state and movie-watching conditions were projected to the polar coordinate space and cortical surfaces (Fig. 6a). The correlation of the group-level sensory magnitudes and sensory angles were presented in Table 1. Sensory magnitudes exhibited similarity across brain states (movie-watching vs. resting-state) and scanning conditions (3 T vs. 7 T). However, sensory angles appeared to be state-dependent, with lower correlations between movie-watching and resting-state but with high correlations across resting-state scans despite being from separate data collections (3 T vs. 7 T).

Given the similarity in sensory magnitudes between movie-watching and resting-state conditions, we focused on the vertex-level comparison of sensory angles. We conducted a paired-sample comparison to investigate the specific areas contributing to differences in sensory angles between the two brain states. Significant between-state angular differences were identified in the fusiform face complex (FFC), superior temporal gyrus (STG), and middle temporal MT+ region (MT, MST, and V4t) (Fig. 6b).

The group-averaged distribution patterns of the STG, MT+, and FFC are illustrated in the polar plots of Fig. 6c. Compared to resting-state, the pattern of the STG cluster shifts from the somatosensory to the auditory domain, while the patterns of the MT+ and FFC shift from the somatosensory to the visual domain. To investigate how the proportional contributions of sensory components influence the above differences, we performed paired-sample comparisons of sensory parameters (β_V_ for visual, β_S_ for somatosensory, and β_A_ for auditory) between movie-watching and resting-state. For each cluster, we calculated the mean parameter for each sensory modality and brain state, and then conducted paired-sample t-tests to separately compare the mean sensory parameters between the two brain states for each sensory modality (see Table 2 and Fig. 6c-boxplots). The comparisons of sensory parameters across states align with the observed pattern transitions in all three clusters. The sensory parameter contributing most to the transition pattern in the STG cluster is β_A_, whereas in the MT+ and FFC clusters, it is β_V_.

**Table 2:** Between-state and between-hemispheric comparisons

Between-state comparisons									
	mean angle			β_V_		β_S_		β_A_	
	MV	RS	p	t	p	t	p	t	p
STG (L)	242.2°	143.5°	0.0	2.7	0.0076	−26.5	1.3e−61	45.1	4.7e−95
STG (R)	244.7°	147.3°	0.0	3.6	0.0005	−25.7	9.6e−60	40.7	2.4e−88
MT+ (L)	12.9°	92.9°	0.0028	27.1	8.8e−63	−23.8	1.7e−55	2.5	0.014
MT+ (R)	14.3°	94.6°	0.0016	33.1	6.0e−75	−23.4	2.4e−54	−0.4	0.067
FFC (L)	1.2°	84.9°	0.0134	36.8	1.2e−81	−17.4	2.2e−39	4.5	1.3e-05
FFC (R)	7.8°	94.1°	0.006	36.3	6.2e−81	−17.6	9.5e−40	0.9	0.035

Between-hemispheric comparisons									
	mean angle			β_V_		β_S_		β_A_	
	L	R	p	t	p	t	p	t	p
55b	293.9°	4.6°	0.0076	−15.9	3.0e−35	−1.7	0.09	19.2	7.1e−44
RI/PSL	210.5°	140.5°	0.0	−4.5	1.3e−05	−24.4	1.2e−56	29.2	2.5e−67
STV	53.6°	82.6°	0.0114	10.5	9.1e−20	−12.9	8.1e−27	2.5	0.014
44/45	332.2°	2.9°	0.0	−8.7	4.2e−15	−4.3	3.2e−05	11.6	2.8e−23
LIPv	265.0°	336.9°	0.0218	−16.3	3.5e−36	−0.5	0.65	13.9	1.5e−29

*β*_*V*_ visual parameter, *β*_*S*_ somatosensory parameter, *β*_*A*_ auditory parameter, *MV* movie-watching, *RS* resting-state, *STG* superior temporal gyrus, *MT* middle temporal, *FFC* fusiform facial complex, *L* left hemisphere, *R* right hemisphere, *RI* retroinsular cortex, *PSL* perisylvian language area, *STV* superior temporal visual, *LIPv* lateral intraparietal ventral.

### Functional relevance

Higher-order cognitive functions may relate to the convergence of sensory information^20^. To explore how sensory integration contributes to the emergence of cognitive functions, we investigated the functional relevance of our model both by mapping specialization using a meta-analytic approach and by characterizing hemispheric lateralization.

**Fig. 7:**
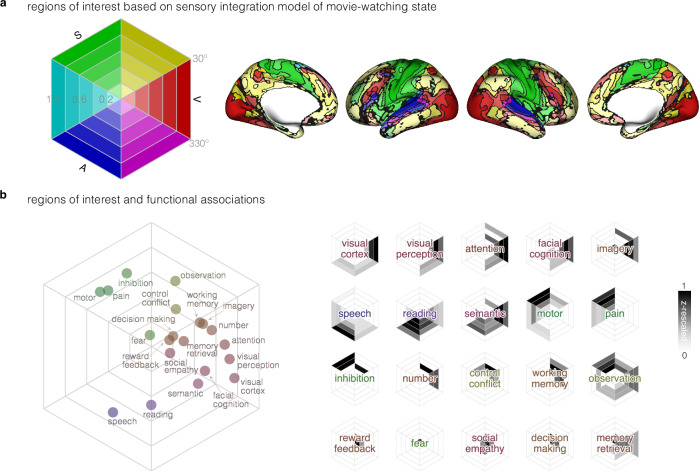
Functional decoding for the sensory integration model of movie-watching. **a** Sensory integration model was divided into 30 ROIs based on evenly divided angles and magnitudes. Regions belonging to each ROI were displayed on the cortical surface; **b** Functional decoding z-values combined with sensory and hierarchical weights projected functional topic terms into a hexagonal coordinate space (left). Small hexagons on the right side illustrated the association of ROIs with each functional term.

To conduct a meta-analysis, it was first necessary to delineate ROIs. We segmented the sensory integration model under the movie-watching condition into 30 regions-of-interest (ROIs) based on six evenly divided angles and five evenly divided magnitudes (Fig. 7a, left). The spatial distribution of ROIs is displayed on the cortical surface using the same coloring scheme based on primary sensory areas (Fig. 7a, right). For each topic, to delineate the association of each ROI with this given topic, the z-values above the threshold (z > 2.327) were rescaled to a range from 0 to 1 and visually represented within the hexagons (Fig. 7b, right). To summarize the distribution of these functional topics in the sensory integration model space, we needed to determine a general location for each topic term under a common coordinate space (Fig. 7b, left).

A darker color in the small hexagons (Fig. 7b, right) indicates a stronger association with that respective topic. A good alignment is observed between the brain function and dimensions of our model. For example, ‘speech’ was primarily linked to auditory regions but also shows connections to the somatosensory and visual domains. For comparison, ‘reading’ was predominantly associated with auditory regions and displayed more connections to the visual domain than the somatosensory domain.

The functional topic terms are sensibly positioned in the left hexagon plot of Fig. 7b, with higher-order functions located at the core of the hexagon and primary functions at the periphery. Topics predominantly related to vision, such as ‘visual perception,’ are situated near the center of the visual domain; the somatosensory-dominated topics, such as ‘motor,’ are positioned close to the somatosensory center; and the auditory-dominated topics, such as ‘speech’, are located around the auditory center. Functions related to multisensory experiences, such as ‘observation,’ are placed within the integrated visual-somatosensory domain (30°-90°), while topics involving the integration of visual or auditory information, such as ‘semantic’ or ‘reading’ are located near the borders of the auditory-visual domain (270° - 330°). Compared to above functions, topics related to higher-order functions, such as ‘social empathy’ or ‘reward feedback’, are situated at the core of the hexagon.

As an analysis to evaluate the functional relevance of the sensory integration model, we next investigated hemispheric asymmetries. Functional lateralization is recognized in several well-studied systems, such as language^29,30^ and spatial processing^31^, providing a test case to evaluate the sensitivity of our model to the known lateralization of these higher-order systems.

**Fig. 8:**
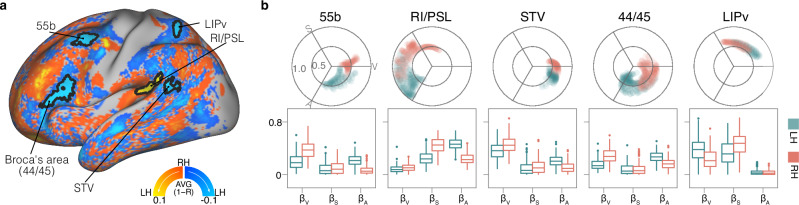
Between-hemispheric comparison of sensory angles under the movie-watching condition. **a** The cortical map displays the group-averaged difference in sensory angles between corresponding locations in the two hemispheres. Areas outlined in black indicate significant differences between the hemispheres. Warm colors represent anticlockwise changes in the angle of the left hemisphere relative to the right hemisphere, while cool colors denote the opposite. **b** Polar plots illustrate the distribution patterns of regions with significant between-hemispheric differences in the sensory integration model, while box plots depict the comparisons of the averaged sensory parameters within each region. For the boxplots, the middle line represents the median, while the box’s upper and lower limits correspond to the 75th and 25th percentiles, respectively. The whiskers extend up to 1.5 times the interquartile range from the upper and lower box limits. Any data points beyond the whiskers are considered outliers and plotted as individual points. The labels V, S, and A mark the anchoring angles for visual (0°), somatosensory (120°), and auditory (240°) modalities. STV superior temporal visual, RI retroinsular cortex, PSL perisylvian language area, LIPv lateral intraparietal ventral, LH left hemisphere, RH right hemisphere.

In contrasting the angles between the left and right hemispheres during the movie-watching condition, significant differences were found in the area 55b, retroinsular cortex and perisylvian language area (RI/PSL), lateral intraparietal ventral area (LIPv), Broca’s area (44/45) and superior temporal visual (STV) (Fig. 8).

It has been well-established that human language functions are predominantly lateralized to the left hemisphere. Our findings indicated that the between-hemispheric differences in angular distribution are more pronounced for language-related clusters (area 55b, 44/45, RI/PSL and STV) than others. Moreover, the patterns of these clusters on the left hemisphere exhibited more proximity to the auditory domain compared with their counterparts on the right, which likely reflects the language lateralization of these clusters during movie-watching.

Regarding the LIPv cluster, the angular distribution between the two hemispheres exhibited a significant statistical difference despite their proximity and partial overlap. LIPv exemplified task-specific functional lateralization in visuomotor motor processing, with its left hemispheric patterns more proximate to the visual domain.

## Discussion

In this study, we use functional MRI data to develop a model which describes the integration of sensory signals along the processing hierarchy through two dimensions. Sensory magnitude measures the percentage of information explained by primary sensory signals, with its ranks providing a proxy for the sensory processing hierarchy. Sensory angle quantifies the proportional contributions of different sensory components, offering insight into multimodal integrative attributes at specific hierarchical levels. Our model effectively captures shifts in processing modes across distinct brain states and provides a meaningful framework to contextualize different cognitive functions in relation to sensory processing.

### Primary sensory regions as anchors of cortical organization

Sensory processing within the cerebral cortex begins in primary sensory regions and culminates in transmodal representations that are essential for higher-order cognition^19,20^. Recent research has emphasized the importance of the primary cortex in shaping functional connectivity dynamics^32^, further supporting a long line of research showing that the primary sensory cortex anchors the spatial arrangement of brain areas^19,33–36^. The role of primary areas in cortical organization is further supported by observations of the relationship between the geodesic distance along the cortical surface and functional connectivity, which demonstrate that regions located farther from primary areas exhibit more distant functional connectivity^37–39^. Regions of the default mode network (DMN), representing the top level of a hierarchy reflected by the principal connectome gradient, show both the greatest distance and equidistance from primary landmarks—the other end of the hierarchy^8^.

The positioning of the primary sensory cortex at an opposite end to association areas, as highlighted in functional connectome studies^7,8^, is corroborated by various sources, including histology^1,2^, functional task activation patterns^40^, cortical microstructure^3–6^, neurotransmitter^41^, and gene expression^42^. Our model captures the same positioning mode. As depicted in Fig. 5, following the sensory propagating stream from primary to association areas, the spatial pattern shifts along an increasing hierarchical axis from the periphery to the core. The consistent trend across these different cortical gradients underscores the critical anchoring role of the primary sensory cortex in cortical organization.

### Sensory hierarchy as the fundamental principle of cortical organization

The gradients discussed above confirm the existence of a dominant axis in cortical organization, indicating a hierarchy extending from lower- to higher-level regions. As a fundamental organizing principle, hierarchy serves as a framework for signal transmission^9–11^, making it an essential dimension to be addressed by our model. It is noteworthy that despite an overarching trend across multiple spatially similar gradients, an increasing number of studies suggest differences among them^43,44^. Rather than incorporating known gradients for mapping the hierarchy, our focus lies on the hierarchy of sensory processing which aligns with the objective of our model. Given that representations become progressively more abstract as signals propagate from primary sensory to higher-order areas^12,13^, we used the percentage of variance explained by the primary sensory signal to signify the abstractness of sensory information and their ranks to represent hierarchical orders of sensory processing. Notably, the sensory magnitude (the dimension of our model representing the rescaled ranks) exhibited a strong similarity to the principal connectome gradient which has been widely accepted as a representation of the functional hierarchy (Fig. 3). The consistent hierarchical trend was further demonstrated by between-session correlations of magnitudes (Table 1, Fig. 4), highlighting the stability of hierarchical orders across diverse conditions. This stable unimodal-to-transmodal hierarchical order even persisted in disease cohorts showing a compressed hierarchy^45–47^. The explanation for this compression remains controversial, however, gaining insight into it holds the potential to significantly advance our understanding of the stability and flexibility of the human brain. Here we used the proportion of sensory components to characterize functional features at specific hierarchical levels.

### Sensory proportion as a state-dependent measure of shifting brain activity pattern

In contrast to hierarchy, the proportional contributions of difference sensory modalities, which was characterized by sensory angels, exhibit consistent patterns exclusively within the same brain state, even under different scanning conditions (Table 1, Fig. 4), rendering it a suitable state-dependent measure.

The movie-watching task paradigm required participants to process diverse, dynamic visual and auditory stimuli compared to the resting-state, leading to different patterns of functional organization^23,48–53^. The decomposition of the movie-watching connectome resulted in three top hierarchical gradients specific to sensory modalities (sensorimotor, visual, auditory/language)^23^, distinct from the resting state^8^. This suggests that while sensory information continues to be transmitted and processed along a stable hierarchy, the proportion of sensory modalities may change depending on contextual demands. This assertion is further supported by the comparison of sensory angles between movie-watching and resting-state.

In response to the heightened demand for visual information processing during movie-watching, the spatial patterns of the fusiform face complex (FFC, functioning for facial recognition)^54–56^ and the middle temporal area (MT + , functioning for visual motion processing)^57,58^, shift towards visual-dominant areas compared to their patterns during the resting-state condition. Similarly, a parallel trend is observed in response to increased auditory processing demands during movie-watching, the spatial pattern of the superior temporal gyrus (STG), associated with sound and language processing^59–61^, shifts towards the auditory-dominant area relative to the resting-state pattern. Notably, the three areas above were included in recurrent patterns of brain activation responding to the cognitive demands of naturalistic paradigms based on a large-scale meta-analysis^62^. And STG showed a remarkably high difference between movie-watching and resting-state in the connectome decomposition space^23^. In addition to the state-dependent shift, functional lateralization was also identified through the sensory proportion analysis. The pronounced differences between the two hemispheres were language-related regions, including Broca’s area (44/45), RI/PSL, STV and area 55b. The task-specific lateralization of the visuomotor was also detected. These findings underscore the crucial role of sensory proportion in unraveling the intricate relationship between cognitive functions and sensory integration.

### Functional relevance of sensory integration mapping

To further illustrate the connection between cognitive functions and the sensory integration model, a series of functional topic terms were placed under the same space of the sensory integration model. The coordinate of each functional term was calculated based on this functional topic’s associations with 30 ROIs generated through an arbitrary thresholding on the sensory integration model.

Notably, this arbitrary procedure for generating ROIs resulted in a parcellation of the cortical surface that displayed broad similarities with other cortical atlases. While the borders of specific areas vary, the general orientation and arrangement of divisions remains consistent with classical architectonic-based atlases^63–66^ as well as more recent connectivity-based cortical atlases^22,24^. This observation supports the intriguing interpretation of primary areas having an anchoring role in cortical differentiation. This notion is central to theories such as grounded cognition^67^, in which abstract forms of cognition and representation emerge from building blocks in sensory experience. The notion that the specialization within the cerebral cortex may also be guided based on the elementary geometries of primary areas may provide an intriguing avenue for future research.

### General applications

The process by which information from unimodal systems is integrated to form abstract and multimodal representations is crucial for multiple aspects of higher-order cognition^19,20^. The model proposed in this study captures adaptive sensory integration along the cortical hierarchy and facilitates establishing a connection between multisensory integrative patterns and cognition. Notably, this model is not limited to visual, somatosensory, and auditory integration but can be extended to encompass other forms of integration such as semantic concepts or higher-order sensory representations.

Evolutionary changes exhibit a decreasing gradient starting from the unimodal cortex and reaching its apex in the posterior regions of the DMN. In other words, humans and macaques are more similar in unimodal regions and less similar in higher-order areas^68^, which indicates a different sensory processing mode across species. Our model, which captures key features of sensory processing organization, provides a potential to gain insights into how evolution shapes human cortical organization. In addition, brain functions in infancy are characterized by prevalent short-range connectivity, whereas long-range network connections become increasingly prominent with age^69–76^. It suggests a developmental shift from a locally to a globally distributed spatial framework^77^. The model presented here may help to further inform how sensory processing modes change along the lifespan.

Our model may also be applicable to clinical research. Sensory integration disorder (SID) is an inability to perform normal sensory processing, adversely affecting learning, coordination, behavior, language, and sensorimotor development, and impeding daily activities and occupational participation^78–80^. Aspects of sensory processing have also been implicated in multiple neurodevelopmental disorders including Autism Spectrum Disorder, Attention-Deficit Hyperactivity Disorder, Developmental Coordination Disorder, and learning disabilities such as dyslexia^81–83^. Our model could aid in the identification and potential interventions for individuals affected by these disorders. Moreover, our model is particularly relevant to individuals experiencing the absence of one or more sensory modalities. It provides a robust framework for investigating the nature and mechanisms of sensory reorganization in such cases. By quantifying the adaptive patterns of sensory convergence in the absence of specific sensory inputs, our model enables a detailed mapping of how the brain compensates and adjusts its sensory integration processes. Furthermore, the model serves as an effective measure to pinpoint aberrations in sensory integration that may contribute to various cognitive deficits. By identifying specific points of abnormal integration, it aids in delineating the neural underpinnings of cognitive impairments, paving the way for targeted interventions, and tailored therapeutic approaches to address these deficits.

### Limitations

One key assumption of our method is the reliance on primary sensory areas as the anchors of cortical organization. While this selection of ROIs was supported by data-driven studies^23^, future research may nevertheless benefit from loosening this constraint. Additionally, the treatment of equal sensory parameters in our model, addressed by assigning them a value of zero, presents a constraint for special cases that may require further consideration in future work. However, it should be noted that in the current study no cortical vertices demonstrated identical sensory parameter values. Finally, the model’s current design, tailored for capturing the integration of three main sensory modalities, poses limitations when applied to higher-order functions that involve the integration of more than three modalities. Future research efforts could prioritize refining the model or developing extensions to broaden its scope, ensuring its efficacy in investigating complex sensory integrative scenarios. In sum, recognizing and addressing these limitations will improve the model’s applicability and accuracy in future work.

In conclusion, a function-based mapping of sensory integration along the cortical hierarchy provides a framework for characterizing cortical organization based on sensory processing. This framework supports the foundational role of the cortical hierarchy for stable cognitive operations and emphasizes the significance of adaptive multisensory integration for flexible responses to contextual demands. Our framework integrates the stability and flexibility inherent in cortical organization and presents an alternative perspective for unraveling the intricacies of cognition through sensory information processing. While other modalities such as cortical architectonics, cross-species comparative anatomy, connectivity, and gene expression may help in establishing an intrinsic space relevant for mapping cortical organization, here we focused on functional attributes with respect to sensory information. Future work integrating across these modalities may hold promise for understanding the emergence and alignment of higher cognitive functions across species, individuals, and the lifespan.

## Methods

### MRI data

The MRI data used in this study were sourced from the Human Connectome Project (HCP)^84^. The dataset included 184 subjects who participated in both movie-watching (7 T) and resting-state scans (7 T and 3 T). We focused on 167 subjects who completed all four runs of movie-watching scans (7 T) and both types of resting-state scans (7 T and 3 T), with each subject contributing an equal number of volumes for each kind of scan session. The participant cohort, consisting of 101 females and 66 males, are all healthy young adults (mean age = 29.4 years, SD = 3.24 years). The recruitment procedures and informed consent forms for participants were approved by the Washington University Institutional Review Board (IRB) as part of the HCP.

The HCP 7 T fMRI data were acquired on a 7 Tesla Siemens Magnetom scanner using the following parameters: 1.6-mm isotropic voxels, repetition time (TR) = 1000 ms, echo time (TE) = 22.2 ms, flip angle = 45°, field of view (FOV) = 208 × 208 mm, matrix = 130 × 130, number of slices = 85, multiband factor = 5, echo spacing = 0.64 ms, and bandwidth (BW) = 1924 Hz/Px. The direction of phase encoding alternated between posterior-to-anterior (PA; MOVIE2, MOVIE3, REST1, and REST3) and anterior-to-posterior (AP; MOVIE1, MOVIE4, REST2, and REST3) across runs.

The HCP 3 T fMRI data were acquired on a 3 Tesla Siemens Connectom Skyra scanner with the following parameters: 2.0-mm isotropic voxels, TR = 720 ms, TE = 33.1 ms, flip angle = 52°, FOV = 208 × 180 mm, matrix = 104 × 90, number of slices = 72, multiband factor = 8, echo spacing = 0.58 ms, and BW = 2290 Hz/Px. The phase encoding direction alternated between right-to-left (RL) and left-to-right (LR) across runs.

Throughout the movie-watching sessions, participants passively viewed video clips featuring audiovisual content. Each session comprised 4 or 5 clips, interspersed with 20-second rest intervals. MOVIE1 (921 TRs) and MOVIE3 (915 TRs) incorporated clips sourced from various independent films, encompassing both fictional and documentary genres, and freely accessible under the Creative Commons license on Vimeo. MOVIE2 (918 TRs) and MOVIE4 (915 TRs) comprised clips sourced from Hollywood films. The presentation format involved a full-screen display, and audio was conveyed through Sensitometric earbuds.

Throughout the resting-state scans, participants were directed to keep their eyes open and sustain a relaxed focus on a bright crosshair displayed against a dark background. Each run comprised 900 TRs in the 7 T dataset and 1200 TRs in the 3 T dataset.

### Modeling sensory integration

The MRI data underwent processing via the HCP’s minimal preprocessing^85^ and ICA + FIX denoising^86,87^ pipeline. Intersubject registration was enhanced using Multimodal Surface Matching Registration (MSMALL)^88,89^. The preprocessed data were then represented on the standard HCP fs_LR 32k surface mesh, comprising 59,412 nodes excluding the non-cortical medial wall.

Subsequent to the initial processing steps, additional smoothing and standardization procedures were implemented before concatenating runs of identical conditions. Given the 2 mm Full Width at Half Maximum (FWHM) smoothing applied during the HCP’s minimal preprocessing, an extra smoothing step was undertaken to achieve an overall smoothness of 4 mm FWHM via the HCP Workbench^90^ -cifti-smoothing command. The degree of supplementary smoothing was determined as the square root of the difference between 4^2^ and 2^2^.

To reduce the influence of the rest intervals in the movie-watching data, we discarded the resting volumes from each movie-watching run. To account for the hemodynamic delay, we also removed the ten volumes immediately following the resting period. Subsequently, all smoothed data were temporally standardized by subtracting the mean and dividing by the standard deviation (SD) for each time series. The four 7 T movie-watching runs, four 7 T resting-state runs, and four 3 T resting-state runs were then concatenated separately.

To map sensory integration, the contributions of different sensory modalities to each fMRI signal were initially quantified using a general linear model (GLM) with non-negative constraints. The model uses the averaged time series from the primary visual cortex (V1), primary somatosensory cortex (S1), and primary auditory cortex (A1) as predictors for the time series of each vertex. The primary sensory areas were delineated using Glasser’s MMP parcellation^22^, which was produced based on high-quality multimodal data from the same dataset (HCP) used in this study. Collinearity among these primary sensory signals was assessed by calculating their Variance Inflation Factors (VIF), as presented in Supplementary Table 1 and Supplementary Fig. 1. As a parcellation involves functional contributions and is represented at the identical surface space as the data we used, it is the best choice for delineating the primary sensory cortex (the anchor of our model). In this parcellation, parcel V1 corresponds to the primary visual cortex, parcel A1 to the primary auditory cortex, and parcel 1, 2, 3a, and 3b to the primary somatosensory cortex.

The GLM is represented by the following equation,

1 $$\begin{array}{c}Y={\beta }_{V}{t}_{V}+{\beta }_{S}{t}_{S}+{\beta }_{A}{t}_{S}+\varepsilon \\ \begin{array}{c}{minimize}\sum {\left(Y-\left({\beta }_{V}{t}_{V}+{\beta }_{S}{t}_{S}+{\beta }_{A}{t}_{S}\right)\right)}^{2},\\ {subject\; to\; the\; constraint}:{\beta }_{V}\ge 0,{\beta }_{S}\ge 0,{\beta }_{A}\ge 0\end{array}\end{array}$$

*Y* is the dependent variable, i.e., time series of the vertex. *t*_*V*_, *t*_*S*_, and *t*_*A*_ are independent variables, i.e., averaged time series of V1, S1, and A1, separately. *β*_*V*_, *β*_*S*_, and *β*_*A*_ are the non-negative regression coefficients. *ε* is the noise, assumed to be Gaussian-distributed with mean 0, it represents the variance not explained by the primary sensory signal.

The regression coefficients (*β*_*V*_, *β*_*S*_, and *β*_*A*_) obtained from Eq. 1 represent the contribution of each specific sensory modality at each vertex. We refer to these coefficients as “sensory parameters”.

As sensory information ascends the processing hierarchy, its increasing abstraction is attributed to the extraction and transfer of only partial features to higher-order areas. This results in a reduced proportion of variance directly associated with the primary sensory components of the signal.

The following equations calculate the proportion of variance explained within each vertex:

2 $$\begin{array}{c}{{SS}}_{{total}}=\sum {\left(Y-\bar{Y}\right)}^{2};\\ {{SS}}_{\exp }=\sum {\left({Y}_{\!\!{pred}}-\bar{Y}\right)}^{2};\\ {R}^{2}={{SS}}_{\exp }/{{SS}}_{{total}}\end{array}$$

*Y* is the time series of the vertex. $\bar{Y}$ is the globally averaged time series. *Y*_*pred*_ is the time series predicted by primary sensory signals (*t*_*V*_*, t*_*S*_, and *t*_*A*_ in Eq. 1). *SS*_*total*_ means the sum of the squared total variance. *SS*_*exp*_ means the sum of squared variance explained by primary sensory signals. *R*^2^ represents the proportion of variance in *Y* explained by primary sensory signals.

As the association with primary sensory signals diminishes when sensory information propagates from lower- to higher-order regions, we rank the proportion of variance explained by primary sensory signals and rescaled these ranks into a range from 0 to 1, which we term “sensory magnitude”.

To quantify the sensory integration, it is essential to characterize the relationship between different sensory modalities. Drawing inspiration from hue transformation in color science^91^, where RGB values are translated into an angular position within a unit circle to represent their interaction, we adopted a similar approach to translate the sensory parameters (β_V_, β_S_ and β_A_), which capture the contributions of the three sensory modalities, into a single angle. We refer to this angle as the “sensory angle”, the other dimension of the sensory integration model.

The transformation from sensory parameters to an angle are given by:

3 $$\begin{array}{c}{C}_{{\max }}=\,{\max }({\beta }_{V},{\beta }_{S},{\beta }_{A});\\ {C}_{{\min}}={\min}({\beta }_{V},{\beta }_{S},{\beta }_{A});\\ \Delta ={C}_{{\max }}-{C}_{{\min}};\\ Hue=\left\{\begin{array}{ll}\quad\quad\quad\quad\quad{0}^{\circ }, & \Delta =0\\ {0}^{\circ }+{60}^{\circ } \, \, \frac{{\beta }_{S}-{\beta }_{A}}{\Delta }, & {C}_{{\max }}={\beta }_{V}\\ {120}^{\circ }+{60}^{\circ } \, \, \frac{{\beta }_{A}-{\beta }_{V}}{\Delta }, & {C}_{{\max }}={\beta }_{S}\\ {240}^{\circ }+{60}^{\circ } \, \, \frac{{\beta }_{V}-{\beta }_{S}}{\Delta }, & {C}_{{\max }}={\beta }_{A}\end{array}\right.\end{array}$$

*C*_*max*_ is the maximum sensory parameter. *C*_*min*_ is the minimum sensory parameter. ∆ is the difference between *C*_*max*_ and *C*_*min*_. Hue is the angular position in a unit circle with a range from 0° to 360°.

We employed the polar coordinate system to visualize our sensory integration model (Fig. 1c). In this system, the sensory angle represents the angular dimension, and sensory magnitude corresponds to the radial dimension. The color encoding is a reverse RGB (Red, Green, and Blue) mapping of HSV (Hue, Saturation, and Value) colors, with sensory angle as hue, sensory magnitude as saturation, and a predefined constant of 0.86 as brightness (which is called value in HSV color model).

### Test-retest reliability

To assess the reliability of the sensory angle and sensory magnitude, we split functional runs for each brain state into test and retest sessions. In the case of movie-watching data at 7 T, the concatenation of MOVIE1 and MOVIE3 comprised the first session. The clips watched during this session were independent films. Subsequently, MOVIE2 and MOVIE4 were concatenated as the second session and clips viewed during this session were Hollywood films. For 7 T resting-state data, the concatenation involved REST1 and REST2 as the first session and REST4 and REST3 as the second session. In the context of 3 T resting-state data, the concatenation incorporated REST1_LR and REST2_RL as the first session, while REST1_RL and REST2_LR constituted the second session.

We calculated between-session correlations for sensory angles using circular correlation^92^ and for sensory magnitudes using Spearman rank correlation.

### Spatial patterns along sensory streams

To investigate the validity of the sensory integration model, we evaluated its ability to capture known sensory processing streams. Following the sensory stream from lower- to higher-order regions, the pattern should shift from the periphery to the core but stay within relevant sensory domains.

We selected ROIs to capture known sensory processing streams for visual, somatosensory, and auditory modalities. The ventral visual stream consists of the primary visual area (BA17) to the secondary visual area (BA18), then the associative visual area (BA19), fusiform gyrus (BA37), and finally the inferior and middle temporal gyrus (BA20 and BA21)^93,94^. The ventral auditory stream consists of early auditory areas (A1, A2, and A3) to auditory association areas (A4 and A5), and finally the dorsal and ventral superior temporal sulcus (STSd and STSv)^95,96^. The dorsal somatosensory stream consists of the primary somatosensory area (BA1, BA2, and BA3) to the superior parietal lobule (BA5) and visuomotor coordination area (BA7), and finally the supramarginal gyrus (BA40)^97,98^.

### Between-state comparison (movie-watching vs. resting-state)

Compared to the resting-state, the movie-watching paradigm presents a greater variety of visual and auditory stimuli, thereby amplifying the discrimination between various sensory modalities and inducing alterations in sensory integration within visual- and auditory-related areas. We performed statistical comparisons between movie-watching and resting-state using sensory angles and sensory magnitudes to examine the capability of our model in capturing state-dependent differences.

For the global level comparison, we calculated the correlations of the group-level sensory angle and the group-level magnitude between each pair of three datasets (Movie 7 T, Rest 3 T and Rest 7 T). The group-level sensory angle was calculated by averaging all individual angles. As for the group-level sensory magnitude, we ranked the group-averaged proportions of variance explained by primary sensory signal, and then rescaled these ranks to a range from 0 to 1 to obtain the group-level magnitude. Correlations of sensory angles were calculated using circular correlation^92^, and correlations of sensory magnitudes were computed using Spearman rank correlation.

At the vertex level, we performed paired-sample comparisons of individual-level metrics between movie-watching and resting-state conditions under 7 T scanning. The between-state comparison of sensory magnitude was assessed by a paired-sample t-test, while the between-state difference in sensory angles was defined as the variance between the two angles, calculated using the following equations.

4 $$\begin{array}{c}cm=({e}^{i\theta 1}+{e}^{i\theta 2})/2;\\ R=\Vert cm\Vert ;\\ V=1-R\end{array}$$

*θ1* and *θ2* are angles (hues obtained from Eq. 3) of two brain states. cm is the complex mean of two angles *θ1* and *θ2*. *R* is the resultant vector length of two angles within a unit circle. *V* is the variance of two angles, which is equal to measuring their difference.

The brain areas showing significant differences between movie-watching and resting state were located through 95th percentile thresholding and a cluster-based permutation test. The process involved 5000 permutations to determine the clusters to retain after thresholding the group-averaged between-state difference map with 95th percentile. In each permutation, a random number of individual difference maps underwent sign-flipping. The permuted group-averaged difference map was thresholded at the 95th percentile. The maximum cluster size for each permutation was recorded. Subsequently, the cluster-level threshold was set at the 95th percentile of the distribution of permuted maximum cluster sizes.

Furthermore, the impact of specific methodological choices on the between-state comparison was evaluated. First, we examined the effect of removing resting volumes from the movie-watching data by comparing results with and without these volumes (Supplementary Table 2 and Supplementary Fig. 2). Second, the influence of non-negative constraints was tested by comparing outcomes from constrained and unconstrained regression models (Supplementary Table 3 and Supplementary Fig. 3).

### Functional relevance

In what way might higher-order cognitive functions be related to the convergence of sensory processing gradients? Our subsequent objective was to explore the alignment of a diverse range of cognitive functions onto our BOLD-signal based mapping of sensory integration along the cortical hierarchy.

Meta-analytic functional decoding provides a means of predicting functions associated with brain regions using a large-scale meta-analytic database^99^. The sensory integration model was segmented into 30 ROIs, defined by five equally spaced magnitudes (ranging from 0 to 1 with increments of 0.2) and six evenly divided angles (330°-30°, 30°-90°, 90°-150°, 150°-210°, 210°-270°, and 270°-330°). Brain areas corresponding to each ROI were binarized and then projected into volumetric space using the ‘metric-to-volume-mapping’ command in the HCP workbench. The Neurosynth MKDA Chi-squared meta-analytic decoding method derived probabilities for functional inference from the ROIs to functional topics. Beginning with the latent Dirichlet allocation (LDA) 50 topics set, we selected 20 topics by excluding non-functional terms such as ‘age_adults_older’ or ‘asd_autism_group’. Subsequently, we obtained 30 z-values for each topic in the meta-analysis, deriving the functional associations of each ROI.

To ensure the inclusion of only notably high associations, a threshold of 2.327 was applied to the z-values for each topic. To show the relationship between this function and ROIs, we displayed thresholded z-values in a hexagon, corresponding with the six-part angular division used to define the ROIs of our meta-analysis. Finally, to obtain an overall portrait of the functional relevance of the sensory integration model, we projected all function topic terms into a common hexagonal coordinate space.

To determine hexagonal coordinates, we introduced four distinct weights: three sensory weights (visual, somatosensory, and auditory) and one hierarchical weight. These weights were combined with thresholded z-values and applied to 30 ROIs from our model. For each sensory weight, we assigned values based on angular ranges: 1 for the sensory-dominant ranges, 0.5 for sensory-integrative ranges, and 0 for others. For instance, the visual weight was set to 1 for ROIs with an angular range of 330°-30° (visual dominant domain), 0.5 for ROIs with ranges of 30°-90° (visual-somatosensory integrative domain) and 270°-330° (visual-auditory integrative domain), and 0 for other ROIs. Hierarchical weights were assigned as follows: 0.9 for the magnitude range of 0.8-1.0, 0.7 for 0.6-0.8, 0.5 for 0.4-0.6, 0.3 for 0.2-0.4, and 0.1 for 0-0.2.

The calculation of each term’s angle involved multiplying the thresholded z-values by sensory weights, followed by their summation and division by the count of non-zero z-values. The resulting three sensory associations were then transformed into an angle using the hue transformation. For the computation of each term’s magnitude (the level in sensory hierarchy), we initially determined the weight of each ROI based on its z-values, followed by its multiplication with the hierarchical weight.

The resulting values were then transformed as the following equation to fit the hexagon.

5 $$\begin{array}{c}d=\left\lfloor\frac{D}{30}\right\rfloor{mod}\,2;\\ M=\left\{\begin{array}{cc}m\frac{\sqrt{3/4}}{\cos (D\,{mod}\,30)}, & d=0\\ m\frac{\sqrt{3/4}}{\cos (30-(D\,{mod}\,30))}, & d\ne 0\end{array}\right.\end{array}$$

*D* is the angular position of each functional topic in the hexagon. *d* is to determine the angular area where *D* is located to select the calculation of *M*. m is the averaged product of z-values and hierarchical weights. *M* is the magnitude of each functional topic in the hexagon.

A second “test” of the functional relevance of our model is functional lateralization, which has been reported in multiple cognitive domains^29–31,100–102^. Given that our model represents sensory integration that is closely tied to brain function, we hypothesized that interhemispheric differences identified by our model would align with known lateralized cognitive functions. The difference between corresponding vertices in two hemispheres was calculated by the method shown in Eq. 4. The group-averaged cross-hemispheric difference maps were corrected using the same method applied in the between-state comparisons, which included a 5000 times sign-flipping cluster-based permutation test.

### Statistics and reproducibility

Correlations of sensory angles were calculated using circular correlation^92^, and correlations of sensory magnitudes were computed using Spearman rank correlation.

Comparisons of sensory angles were measured by the variance between angles, while comparisons of sensory magnitudes and sensory parameters were conducted using paired-sample t-tests. A significance threshold of *p* < 0.05 was applied to identify brain areas showing significant differences between brain states or hemispheres. Significant areas were located using a 95th percentile thresholding and a cluster-based permutation test. For each significant cluster, its exact significance was determined by calculating the ratio of the number of permutations with a cluster size larger than the observed cluster to the total number of permutations.

Reproducibility was assessed across test-retest concatenated sessions from three conditions (Movie 7 T, Rest 3 T and Rest 7 T) by calculating between-session correlations in sensory angles and sensory magnitudes.

The code used in this study has been publicly released^103^.

### Reporting summary

Further information on research design is available in the Nature Portfolio Reporting Summary linked to this article.

## Supplementary information

Supplementary Information

Reporting summary

## Data Availability

Data were provided by the Human Connectome Project, WU-Minn Consortium (Principal Investigators: David Van Essen and Kamil Ugurbil; 1U54MH091657) funded by the 16 NIH Institutes and Centers that support the NIH Blueprint for Neuroscience Research; and by the McDonnell Center for Systems Neuroscience at Washington University. All data are obtainable from the HCP website (https://db.humanconnectome.org/).

## Code availability

The code (10.5281/zenodo.14045361) for model construction, statistical analysis, and figure making has been made publicly available at https://github.com/K-Z-W/sensory-integration-model.
