# Supplementary material for: A function-based mapping of sensory integration along the cortical hierarchy
Source: Commun Biol. 2024 Nov 29;7:1593. doi: 10.1038/s42003-024-07224-z (PMC11607388; doi:10.1038/s42003-024-07224-z)
Supplement: Supplementary file 1 — Supplementary Information [file 42003_2024_7224_MOESM1_ESM.pdf]

Supplementary material

Collinearity test

To map sensory integration, we quantified the contributions of different sensory modalities to the fMRI signal at each vertex using a general linear model (GLM). The averaged time series from the primary visual cortex (V1), primary somatosensory cortex (S1), and primary auditory cortex (A1) served as predictors for the time series at each vertex. Collinearity among these primary sensory signals can inflate the variance and standard error of the regression coefficients, potentially reducing the reliability of our sensory integration model. To assess collinearity, we calculated the Variance Inflation Factor (VIF) for each predictor, specifically the averaged time series from V1, S1, and A1, for each subject.

VIF quantifies the extent to which the variance of a regression coefficient is increased due to collinearity. For each predictor in a regression, the VIF is computed by regressing this predictor on all other predictors in a separate regression model. The VIF for a predictor is given by:

$$VIF_i = \frac{1}{1 - R_i^2}$$

where R_i^2 is the coefficient of determination from the regression of the *i*th predictor on the other predictors.

Typically, a VIF less than 5 indicates acceptable collinearity in a linear regression. Our results showed that all subjects had low VIFs for the primary sensory signals, indicating minimal collinearity. Therefore, no further orthogonalization procedures were necessary.

Supplementary Table 1. Variance inflation factors for each predictor in different conditions

	3T-resting state		7T-resting state		7T-movie watching	
	mean	std	mean	std	mean	std
TS-V1	1.48	0.37	1.43	0.28	1.37	0.15
TS-S1	1.48	0.35	1.67	0.34	1.22	0.16
TS-A1	1.49	0.33	1.72	0.39	1.38	0.6

TS indicates the averaged time series; V1, primary visual cortex; S1, primary somatosensory cortex; A1, primary auditory cortex.

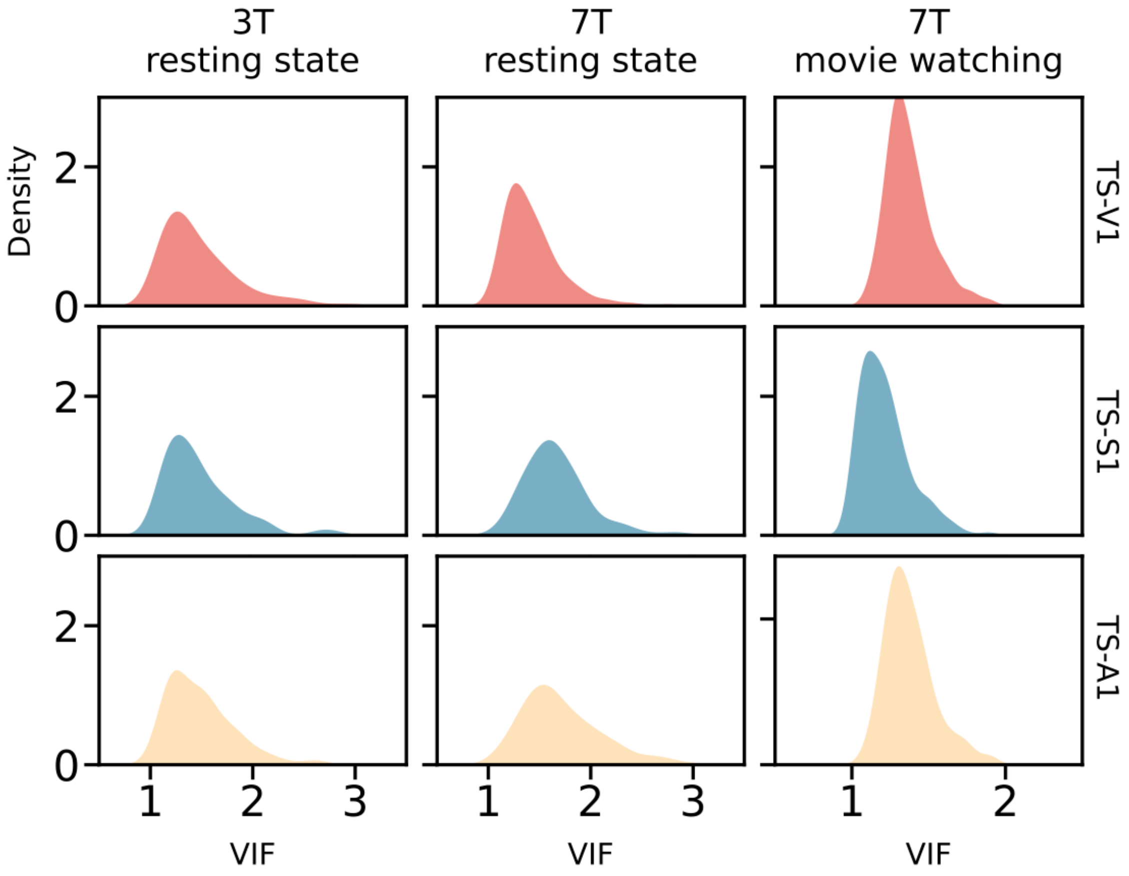

Supplementary Figure 1. Distribution of VIFs for each predictor in different conditions. VIF, variance inflation factor; TS, the averaged time series; V1, primary visual cortex; S1, primary somatosensory cortex; A1, primary auditory cortex.

Movie-watching data including resting volumes

Given that the removal of resting volumes from movie-watching data is not a standard preprocessing procedure, we also provide results using movie-watching data that includes these resting volumes.

At the group-level, the correlation between movie-watching data with and without resting volumes was 0.965 for sensory angles and 0.986 for sensory magnitudes. The distribution of sensory integration mappings was similar between these two data sets on the cortical surface. However, the polar projections of the sensory integration mappings were less consistent (Supplementary Figure 2a).

The correlations of group-level metrics for all conditions are presented in Supplementary Table 2.

Supplementary Table 2. Correlation of group-level metrics between conditions

Correlation of sensory angles between different conditions

	MV-7T (rs_exd)	MV-7T (rs_ind)	RS-7T	RS-3T
MV-7T (rs_exd)	1			
MV-7T (rs_ind)	0.965	1		
RS-7T	0.477	0.378	1	
RS-3T	0.558	0.441	0.907	1

Correlation of sensory magnitudes between different conditions

	MV-7T (rs_exd)	MV-7T (rs_ind)	RS-7T	RS-3T
MV-7T (rs_exd)	1			
MV-7T (rs_ind)	0.986	1		
RS-7T	0.952	0.922	1	
RS-3T	0.914	0.916	0.946	1

MV, movie-watching; RS, resting-state; rs_exd, excluding resting volumes; rs_ind, including resting volumes.

Subsequently, we conducted a vertex-wise comparison at the individual-level between resting-state data and movie-watching data excluding resting volumes. For sensory magnitude, there was no significant difference between the resting-state and movie-watching conditions. As for sensory angle, significant differences in sensory angle were observed between the states in the fusiform face complex (FFC), superior temporal gyrus (STG), and middle temporal MT+ region. Notably, when resting volumes were excluded from the movie-watching data, the 9-

46/46 area was no longer significantly different between the two brain states (Supplementary Fig 2b).

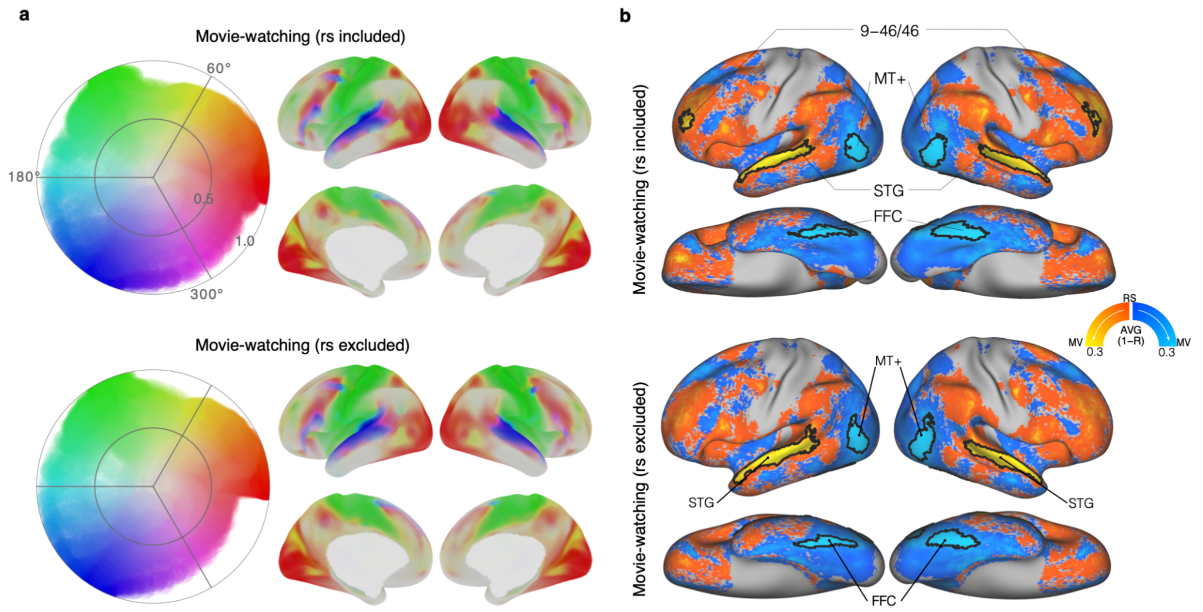

Supplementary Figure 2. Comparison between movie-watching data with and without removing resting volumes. a Sensory integration mapping of movie-watching data with and without removing resting volumes. **b** Between-state (movie-watching vs. resting-state) comparison results by using the movie-watching data with and without removing resting volumes. STG, superior temporal gyrus; FFC, fusiform facial complex; MT, middle temporal; RS, resting-state; MV, movie-watching.

Linear regression without non-negative constraints

We quantified the contributions of different sensory modalities to the fMRI signal at each vertex using a linear regression with non-negative constraints. To thoroughly assess the impact of non-negative constraints, we performed linear regressions without constraints and compared the outcomes to our results using non-negative constraints.

First, we calculated the group-averaged sensory parameters derived from using the unconstrained regression for both movie-watching (7T) and resting-state (7T) data. Then, we visualized their spatial distributions on the cortical surface (Supplementary Figure 3a). We observed that negative sensory parameters were predominantly located in the primary sensory cortex. For instance, during movie-watching, negative values were mainly concentrated in the primary somatosensory and auditory areas. In our regression model, which incorporates three primary sensory signals as predictors, negative sensory parameters may suggest that one sensory modality is overly influential, leading to negative values as a result of optimizing the

prediction—a scenario that does not align with our goal of quantifying the contributions of different sensory modalities.

Second, we examined the distributions of negative and positive sensory parameters derived from using unconstrained regression. We found that the distribution of negative values was relatively narrow and close to zero compared to positive values (Supplementary Figure 3b). Additionally, we assessed the changes in these negative values after applying non-negative constraints. Our analysis revealed that the negative sensory parameters became positive, with an even narrower distribution and values closer to zero (Supplementary Figure 3c).

Third, within areas with negative values, we found a significant overlap between applying and not applying non-negative constraints (Supplementary Figure 3d). We further examined whether applying non-negative constraints would impact our statistical comparisons at the global and vertex level across all values. The correlation of sensory angles between using and not using non-negative constrained regression is 0.987 for movie-watching data and 0.953 for resting-state (7T) data. The correlation of sensory magnitudes is 0.9997 for movie-watching data and 0.996 for resting-state (7T) data. The correlations of group-level metrics for all conditions are presented in Supplementary Table 3.

At the global level, we observed that the distribution patterns were highly similar with and without the constraint (Supplementary Figure 3e). This similarity is further supported by the correlations of group-level metrics across different conditions (Supplementary Table 3).

At the vertex level, the between-state comparisons were performed separately for the conditions of using and not using non-negative constrained regression (Supplementary Figure 3f). We found that the overall patterns and statistical results remained consistent, indicating that applying non-negative constraints had minimal effects on our findings.

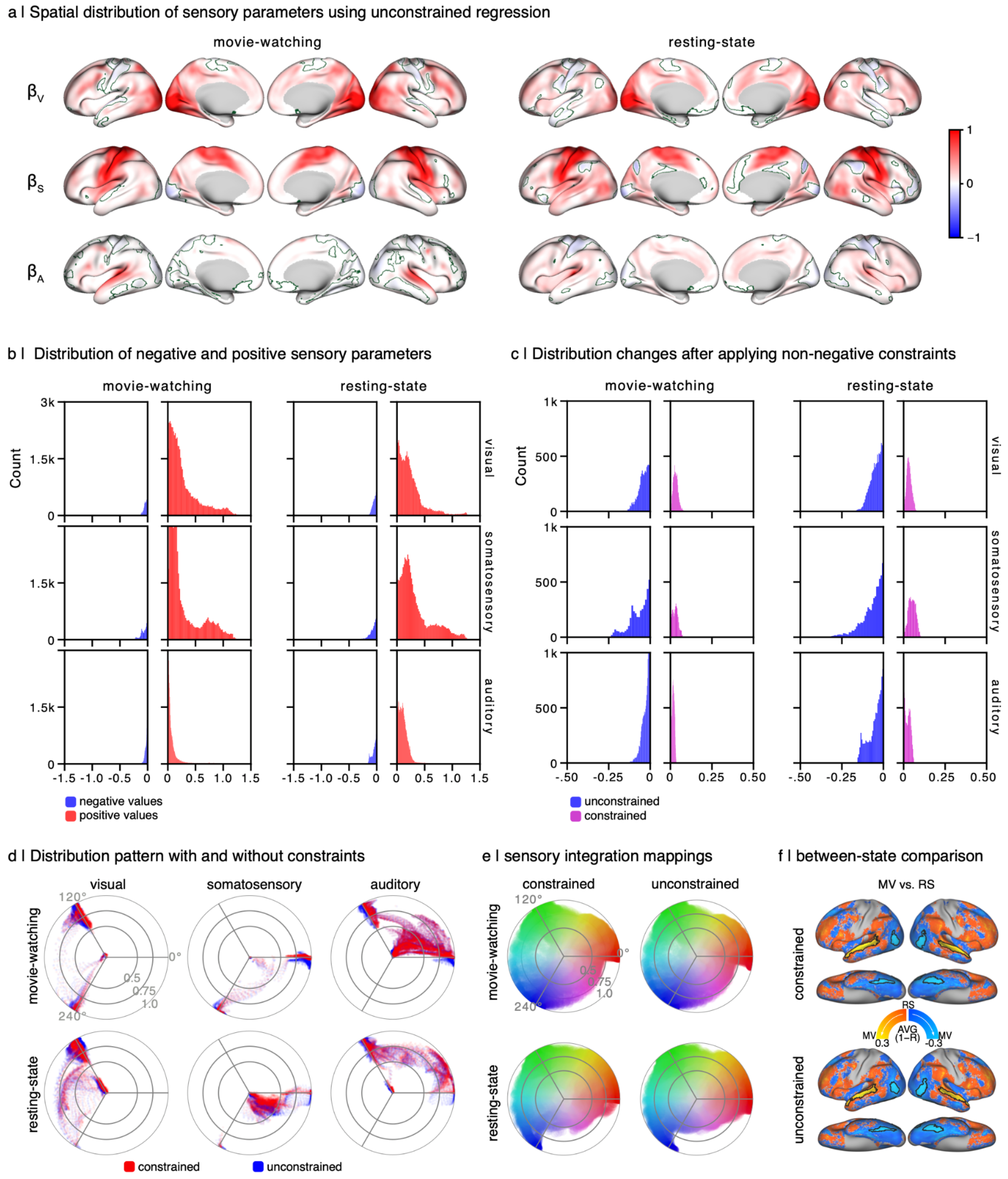

Supplementary Figure 3. Analysis of the effects of non-negative constraints. a Spatial distribution of sensory parameters on the surface in the condition without using non-negative constraints. Negative values are indicated by the green outlines. **b** Distribution of negative and positive sensory parameters in the condition without using non-negative constraints. **c** Changes in distribution of negative sensory parameters after applying non-negative constraints. **d** Distribution patterns of sensory integration polar projection between the conditions of using and not using non-negative constraints. **e** Polar projections of sensory integration mappings in the conditions of using and not using constraints **f** Between-state comparisons in the conditions of using and not using non-negative constraints. MV, movie-watching; RS, resting-state; AVG (1-R), group averaged angular difference which was defined as the resultant vector length R subtracting from 1.

Supplementary Table 3.
Correlation of group-level metrics between using constrained or unconstrained regression

Correlation of sensory angles between using constrained or unconstrained regression

		movie-watching		resting-state	
		constrained	uncocnstrained	constrained	unconstrained
movie-watching	constrained	1			
	unconstrained	0.987	1		
resting-state	constrained	0.477	0.469	1	
	unconstrained	0.452	0.462	0.953	1

Correlation of sensory magnitudes between using constrained or unconstrained regression

		movie-watching		resting-state	
		constrained	uncocnstrained	constrained	unconstrained
movie-watching	constrained	1			
	unconstrained	0.9997	1		
resting-state	constrained	0.951	0.952	1	
	unconstrained	0.954	0.954	0.996	1
