# Supplementary material for: A function-based mapping of sensory integration along the cortical hierarchy
Source: Commun Biol. 2024 Nov 29;7:1593. doi: 10.1038/s42003-024-07224-z (PMC11607388; doi:10.1038/s42003-024-07224-z)
Supplement: Supplementary file 2 — Reporting summary [file 42003_2024_7224_MOESM2_ESM.pdf]

nature portfolio

Corresponding author(s): Wei Wei; Daniel S. Margulies

Last updated by author(s): Nov 1, 2024

Reporting Summary

Nature Portfolio wishes to improve the reproducibility of the work that we publish. This form provides structure for consistency and transparency in reporting. For further information on Nature Portfolio policies, see our Editorial Policies and the Editorial Policy Checklist.

Statistics

For all statistical analyses, confirm that the following items are present in the figure legend, table legend, main text, or Methods section.

n/a	Confirmed	
☐	☒	The exact sample size (*n*) for each experimental group/condition, given as a discrete number and unit of measurement
☐	☒	A statement on whether measurements were taken from distinct samples or whether the same sample was measured repeatedly
☐	☒	The statistical test(s) used AND whether they are one- or two-sided<br>*Only common tests should be described solely by name; describe more complex techniques in the Methods section.*
☐	☒	A description of all covariates tested
☐	☒	A description of any assumptions or corrections, such as tests of normality and adjustment for multiple comparisons
☐	☒	A full description of the statistical parameters including central tendency (e.g. means) or other basic estimates (e.g. regression coefficient) AND variation (e.g. standard deviation) or associated estimates of uncertainty (e.g. confidence intervals)
☐	☒	For null hypothesis testing, the test statistic (e.g. F, t, r) with confidence intervals, effect sizes, degrees of freedom and P value noted<br>*Give P values as exact values whenever suitable.*
☒	☐	For Bayesian analysis, information on the choice of priors and Markov chain Monte Carlo settings
☒	☐	For hierarchical and complex designs, identification of the appropriate level for tests and full reporting of outcomes
☒	☐	Estimates of effect sizes (e.g. Cohen's *d*, Pearson's *r*), indicating how they were calculated

Our web collection on statistics for biologists contains articles on many of the points above.

Software and code

Policy information about availability of computer code

Data collection	No software was used in the data collection process. We downloaded fMRI data included in our study from a public database provided by the Human Connectome Project (HCP).
Data analysis	We used preprocessed fMRI data from HCP. Additional spatial smoothing was conducted using Workbench Command (1.5.0-GCCcore-10.3.0). The model construction, statistical analysis and figures were conducted using our own custom python code, which we have made openly available on GitHub (http://github.com/K-Z-W/sensory-integration-model).

For manuscripts utilizing custom algorithms or software that are central to the research but not yet described in published literature, software must be made available to editors and reviewers. We strongly encourage code deposition in a community repository (e.g. GitHub). See the Nature Portfolio guidelines for submitting code & software for further information.

Data

Policy information about availability of data

All manuscripts must include a data availability statement. This statement should provide the following information, where applicable:

- Accession codes, unique identifiers, or web links for publicly available datasets
- A description of any restrictions on data availability
- For clinical datasets or third party data, please ensure that the statement adheres to our policy

Data were provided by the Human Connectome Project, WU-Minn Consortium (Principal Investigators: David Van Essen and Kamil Ugurbil; 1U54MH091657) funded

by the 16 NIH Institutes and Centers that support the NIH Blueprint for Neuroscience Research; and by the McDonnell Center for Systems Neuroscience at Washington University. All data are obtainable from the HCP website (https://db.humanconnectome.org/)

Research involving human participants, their data, or biological material

Policy information about studies with human participants or human data. See also policy information about sex, gender (identity/presentation), and sexual orientation and race, ethnicity and racism.

Reporting on sex and gender	No sex- and gender-based analyses have been performed in this study. Our study focuses on the group-level representations without specific interest in the demographic-based differences regarding categories such as sex and gender.
Reporting on race, ethnicity, or other socially relevant groupings	No socially relevant groupings have been investigated in this study.
Population characteristics	We included only one cohort of healthy subjects with small age variance (mean age = 29.4 years, SD = 3.24 years).
Recruitment	All data were obtained from a public dataset provided by the Human Connectome Project (HCP)
Ethics oversight	Participant recruitment procedures and informed consent forms were previously approved by the Washington University Institutional Review Board as part of the HCP

Note that full information on the approval of the study protocol must also be provided in the manuscript.

Field-specific reporting

Please select the one below that is the best fit for your research. If you are not sure, read the appropriate sections before making your selection.

☒ Life sciences ☐ Behavioural & social sciences ☐ Ecological, evolutionary & environmental sciences

For a reference copy of the document with all sections, see nature.com/documents/nr-reporting-summary-flat.pdf

Life sciences study design

All studies must disclose on these points even when the disclosure is negative.

Sample size	We did not perform a sample-size calculation. We chose all possible subjects (n=167) who participated in both movie-watching (7T) and resting-state scans (7T and 3T). And the number of volumes for each scan session was consistent across all subjects. Each subject contributed a substantial amount of data: 3669 volumes for movie-watching (across 4 runs), 3600 volumes for resting-state scans at 7T (across 4 runs), and 4800 volumes for resting-state scans at 3T (across 4 runs). This extensive time series data also ensures the generation of stable results given by our sample size.
Data exclusions	Out of the 184 subjects in the HCP database who participated in both movie-watching (7T) and resting-state scans (7T and 3T), we retained only those (n=167) who completed all 4 runs of movie-watching, resting-state scans at 7T, and resting-state scans at 3T. And the number of volumes for each scan session was consistent across all subjects.
Replication	This study included three kinds of functional data (movie-watching at 7T, resting-state at 7T and 3T) from the same participants. Each data type consists of four scans, divided into two concatenated sessions. The test-retest reliability was assessed by calculating the correlation between sessions for the two dimensions of the sensory integration model developed in this study.
Randomization	No randomization was conducted because there was only one group of subjects in this study.
Blinding	Blinding procedures were not relevant to our study because there was only one group of subjects, and all statistical comparisons in this study were within-subject comparison.

Reporting for specific materials, systems and methods

We require information from authors about some types of materials, experimental systems and methods used in many studies. Here, indicate whether each material, system or method listed is relevant to your study. If you are not sure if a list item applies to your research, read the appropriate section before selecting a response.

Materials & experimental systems

n/a	Involved in the study
☒	☐ Antibodies
☒	☐ Eukaryotic cell lines
☒	☐ Palaeontology and archaeology
☒	☐ Animals and other organisms
☒	☐ Clinical data
☒	☐ Dual use research of concern
☒	☐ Plants

Methods

n/a	Involved in the study
☒	☐ ChIP-seq
☒	☐ Flow cytometry
☐	☒ MRI-based neuroimaging

Plants

Seed stocks

Report on the source of all seed stocks or other plant material used. If applicable, state the seed stock centre and catalogue number. If plant specimens were collected from the field, describe the collection location, date and sampling procedures.

Novel plant genotypes

Describe the methods by which all novel plant genotypes were produced. This includes those generated by transgenic approaches, gene editing, chemical/radiation-based mutagenesis and hybridization. For transgenic lines, describe the transformation method, the number of independent lines analyzed and the generation upon which experiments were performed. For gene-edited lines, describe the editor used, the endogenous sequence targeted for editing, the targeting guide RNA sequence (if applicable) and how the editor was applied.

Authentication

Describe any authentication procedures for each seed stock used or novel genotype generated. Describe any experiments used to assess the effect of a mutation and, where applicable, how potential secondary effects (e.g. second site T-DNA insertions, mosiacism, off-target gene editing) were examined.

Magnetic resonance imaging

Experimental design

Design type

We included fMRI data with both task (movie-watching) and resting-state paradigms.

Design specifications

Throughout the movie-watching sessions, participants passively viewed video clips featuring audiovisual content. Each session comprised 4 or 5 clips, interspersed with 20-second rest intervals. MOVIE1 (921 TRs) and MOVIE3 (915 TRs) incorporated clips sourced from various independent films, encompassing both fictional and documentary genres, and freely accessible under the Creative Commons license on Vimeo. MOVIE2 (918 TRs) and MOVIE4 (915 TRs) comprised clips sourced from Hollywood films. The presentation format involved a full-screen display, and audio was conveyed through Sensitometric earbuds.
Throughout the resting-state scans, participants were directed to keep their eyes open and sustain a relaxed focus on a bright crosshair displayed against a dark background. Each run comprised 900 TRs in the 7T dataset and 1200 TRs in the 3T dataset.

Behavioral performance measures

No behavioral performance measures were included from the HCP dataset in the current study.

Acquisition

Imaging type(s)

We used functional MRI data.

Field strength

We used fMRI data under both 7 Tesla and 3 Tesla.

Sequence & imaging parameters

The HCP 7T fMRI data were acquired on a 7 Tesla Siemens Magnetom scanner using the following parameters: 1.6-mm isotropic voxels, repetition time (TR) = 1000 ms, echo time (TE) = 22.2 ms, flip angle = 45°, field of view (FOV) = 208 x 208 mm, matrix = 130 x 130, number of slices = 85, multiband factor = 5, echo spacing = 0.64 ms, and bandwidth (BW) = 1924 Hz/Px. The direction of phase encoding alternated between posterior-to-anterior (PA; MOVIE2, MOVIE3, REST1, and REST3) and anterior-to- posterior (AP; MOVIE1, MOVIE4, REST2, and REST3) across runs.
The HCP 3T fMRI data were acquired on a 3 Tesla Siemens Connectom Skyra scanner with the following parameters: 2.0-mm isotropic voxels, TR = 720 ms, TE = 33.1 ms, flip angle = 52°, FOV = 208 x 180 mm, matrix = 104 x 90, number of slices = 72, multiband factor = 8, echo spacing = 0.58 ms, and BW = 2290 Hz/Px. The phase encoding direction alternated between right-to-left (RL) and left-to-right (LR) across runs.

Area of acquisition

A whole brain scan was used in this study.

Diffusion MRI ☐ Used ☒ Not used

Preprocessing

Preprocessing software

The fMRI data included in this study was preprocessed by HCP minimal preprocessing and ICA+FIX denoising pipeline.

Normalization	All data underwent temporal standardization by subtracting the mean and dividing by the standard deviation.
Normalization template	The preprocessed data were represented on the standard HCP fs_LR 32k surface mesh.
Noise and artifact removal	The fMRI data underwent HCP's minimal preprocessing and ICA+FIX denoising pipeline, ICA components classified as noise were removed.
Volume censoring	No volume censoring was implemented in this study.

Statistical modeling & inference

Model type and settings	We used an univariate analysis.<br>For the first-level analysis, a non-negative general linear model (GLM) produces three sensory beta values by utilizing averaged time series within V1, S1, and A1 to predict the time series of each vertex.<br>For the second-level, we compared the fixed effect representing the between-condition difference in angle. The angle is calculated from a combination of three beta values, and compared between the movie-watching and resting-state conditions.
Effect(s) tested	We tested for differences in sensory angle between movie-watching and resting-state condition and between two hemispheres. No ANOVA or factorial design was used.

Specify type of analysis: ☒ Whole brain ☐ ROI-based ☐ Both

Statistic type for inference<br>(See Eklund et al. 2016)	Vertex-wise statistic was performed.<br>For each vertex, the difference of two angles was defined as their resultant vector length subtracted from 1.
Correction	The brain areas showing significant differences were located through 95th percentile thresholding and a cluster-based permutation test.<br>The process involved 5000 permutations to determine the clusters to retain after thresholding the group-averaged angular difference map with 95th percentile. In each permutation, a random number of individual difference maps underwent sign-flipping. The permuted group-averaged difference map was thresholded at the 95th percentile. The maximum cluster size for each permutation was recorded. Subsequently, the cluster-level threshold was set at the 95th percentile of the distribution of permuted maximum cluster sizes.

Models & analysis

n/a	Involved in the study
☒	☐ Functional and/or effective connectivity
☒	☐ Graph analysis
☐	☒ Multivariate modeling or predictive analysis

Multivariate modeling and predictive analysis	A general linear model (GLM) with non-negative constrains, using the averaged time series within the primary visual cortex (V1), primary sensorimotor cortex (S1), and primary auditory cortex (A1) to predict the time series of each vertex, generated three sensory beta values. We represented sensory integration across the cortical hierarchy using two dimensions derived from associations with primary sensory signal: sensory angle and sensory magnitude. Sensory angle was obtained by converting three sensory betas into an angle representing the relative combination of primary sensory associations. Sensory magnitude was represented using the rescaled rank of the percentage of the variance explained by primary sensory predictors.
